# Timing of Frontal Plane Trunk Lean, Not Magnitude, Mediates Frontal Plane Knee Joint Loading in Patients with Moderate Medial Knee Osteoarthritis

**DOI:** 10.1155/2018/4526872

**Published:** 2018-03-20

**Authors:** Freyja Hálfdanardóttir, Dan K. Ramsey, Kristín Briem

**Affiliations:** ^1^Faculty of Medicine, School of Health Sciences, University of Iceland, Saemundargata 2, 101 Reykjavík, Iceland; ^2^Department of Health Professions Education, D'Youville College, 220 Koessler Administration Building (KAB), 320 Porter Ave, Buffalo, NY 14201, USA

## Abstract

The purpose of this study was to examine the influence of trunk lean and contralateral hip abductor strength on the peak knee adduction moment (KAM) and rate of loading in persons with moderate medial knee osteoarthritis. Thirty-one males (17 with osteoarthritis, 14 controls) underwent 3-dimensional motion analysis, strength testing of hip abductors, and knee range of motion (ROM) measures, as well as completing the knee osteoarthritis outcome score (KOOS). No differences were found between groups or limbs for gait cycle duration, but the osteoarthritis group had longer double-limb support during weight acceptance (*p* < 0.001) and delayed frontal plane trunk motion towards the stance limb (*p* < 0.01). This was reflected by a lower rate of loading for the osteoarthritis group compared to controls (*p* < 0.001), whereas no differences were found for peak KAM. Trunk angle, contralateral hip abductor strength, and BMI explained the rate of loading at the involved knee (*p* < 0.001), an association not found for the contralateral knee or control knees. Prolonged trunk lean over the stance limb may help lower peak KAM values. Rate of frontal plane knee joint loading may partly be mediated by the contralateral limb's abductor strength, accentuating the importance of bilateral lower limb strength for persons with knee osteoarthritis.

## 1. Introduction

Osteoarthritis (OA), knee OA in particular, is a large and growing public health concern. Notable increases in estimated years lived with disability owing to OA has been observed over the last 15 years [1] with concomitant activity limitations projected to increase [2]. Moreover the economic burden associated with knee OA is expected to rise concurrently with the ageing population, resulting in increased demand for primary and revision knee joint arthroplasty [3]. Therefore, emphasis has been focused on investigating whether conservative biomechanical modalities mediate disease progression [4, 5].

Frontal plane trunk movements have recently garnered increased attention, with evidence suggesting patients with severe medial compartment OA exhibit greater ipsilateral peak trunk lean as opposed to persons with less severity [6]. This may be an effective strategy for mediating pain relief [7], evoked by redirecting the ground reaction force more laterally and lowering medial compartment load as inferred by lower external knee adduction moments (KAM) observed among healthy controls [8–10] and persons with medial knee OA [11] during gait. Perhaps this may be an effective strategy, particularly for persons with mild to moderate knee OA, to attenuate KAM magnitudes similar to that of the uninvolved knee and/or controls, despite varus deformity [12–14].

It is during the first 10% of the gait cycle when transitioning from double- into single-limb support and weight acceptance (from initial contact (IC) of the stance limb until contralateral toe-off) when the first peak KAM typically occurs [15]. Yet the rate of loading throughout weight acceptance (WA), as reflected by the positive slope of the KAM profile, has been associated with medial knee joint degeneration, independent from the peak and KAM impulse [16]. Frontal plane pelvic stability and, thereby, trunk and hip joint motion are regulated by the hip abductor muscles. Effective control of hip and pelvis frontal plane movement calls for good eccentric control, not just of the hip abductors on the affected side but also on the contralateral side for stabilizing the pelvis prior to IC. The rate and magnitude of knee loading during WA may therefore be in part mediated by contralateral hip abductor strength, which has not been well studied. While recent studies have investigated the relationship between hip abductor function and gait parameters, none report an association between contralateral hip abductor strength, neuromuscular activation patterns, or ipsilateral peak KAM magnitudes after accounting for body size [17, 18]. A recent meta-analysis on studies of hip strength in people diagnosed with symptomatic knee OA demonstrated weaker isometric and isokinetic hip abduction strength [19]. Contralateral hip abductor strength would affect control of contralateral pelvic drop during stance, and greater pelvic drop has been shown to increase knee adduction moments [20]. Better understanding of the relationship between hip strength, trunk lean, and knee joint loading in people with knee OA may help clinicians design targeted rehabilitation programs for those affected by knee OA. Therefore, the purpose of this study was to examine whether frontal plane knee joint loading was related to temporal-spatial parameters and to determine whether contralateral hip abductor strength mediates loading in addition to trunk lean. We hypothesized that greater trunk lean would be evident in persons with symptomatic moderate medial compartment OA compared to matched controls, and that either ipsilateral or contralateral hip abductor strength might be inversely associated with loading parameters at the index knee of OA patients during the WA phase of gait.

## 2. Materials and Methods

Data for this cross-sectional laboratory study were collected during baseline measurements of patients who had been referred by orthopedic specialists for a fitting and trial treatment with an unloader (valgus) brace [21]. Due to limited number of female patients at the clinic who were inclined to try an unloader brace, a decision was made to include only males in this study. Seventeen male patients (aged 40–60 years) with confirmed medial knee OA, Kellgren Lawrence (KL) grade 2 or 3 radiographic changes [22], and clinical history of pain and functional impairments fulfilled the inclusion criteria. In cases where bilateral radiographic knee OA was diagnosed, the more symptomatic knee denoted the affected one. Patients were excluded if they had joint replacement surgery, periarticular fracture, osteotomy, knee ligament reconstruction, or arthroscopic surgery to any lower limb joint within 6 months of the study. Exclusion criteria also included radiologically confirmed OA in the ankle or hip joints, intra-articular corticosteroid or viscosupplementation injection to either knee joint within 3 months of study participation, or any musculoskeletal or neurological impairment, dermatological or circulatory problems in the lower extremities that might affect ambulation. Fourteen asymptomatic male subjects were recruited from the university community to serve as controls (CTRL) and were matched to the OA cohort by age (to within 5 years), weight (to within 5 kilograms), and height (to within 5 centimeters).

**Figure 1:**
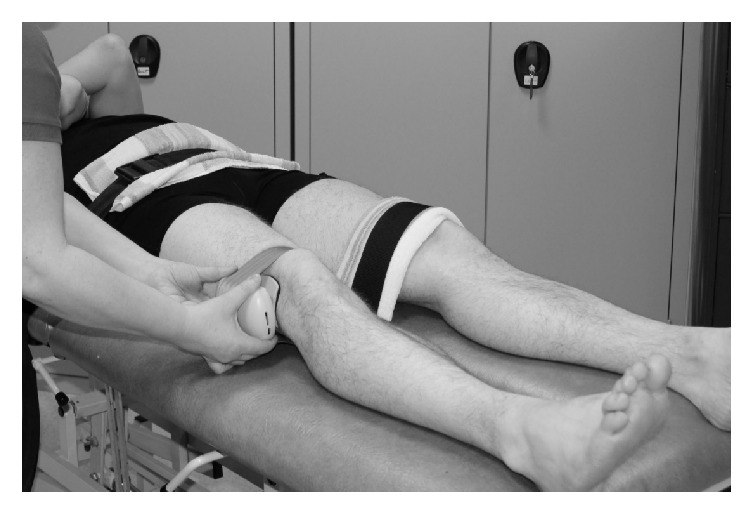
Isometric strength testing of hip abductors. Straps at pelvis and contralateral limb, as well as fixating hand-held dynamometer.

The protocol of this study was approved by the National Bioethics Committee and Data Protection Authority (VSNb2011100025/03.07). Participants signed an informed consent form and then completed the knee osteoarthritis outcome score (KOOS) questionnaire [23, 24]. Passive knee joint range of motion (ROM) was measured bilaterally from full extension to flexion by a single physical therapist with over 10 years of clinical experience. The same physical therapist also conducted hip abductor isometric strength testing with a hand-held dynamometer (Lafayette Manual Muscle Tester, Lafayette, USA) placed 5 cm proximal to the lateral femoral condyle with participants laying supine [25]. Straps were used to secure the pelvis and each lower limb during strength testing (Figure 1). After a single, submaximal practice trial, participants performed three maximal trials of 5 s duration, separated by 15 s of rest. The strongest trial was used for analysis, normalized by the participant's body mass index (BMI).

**Figure 2:**
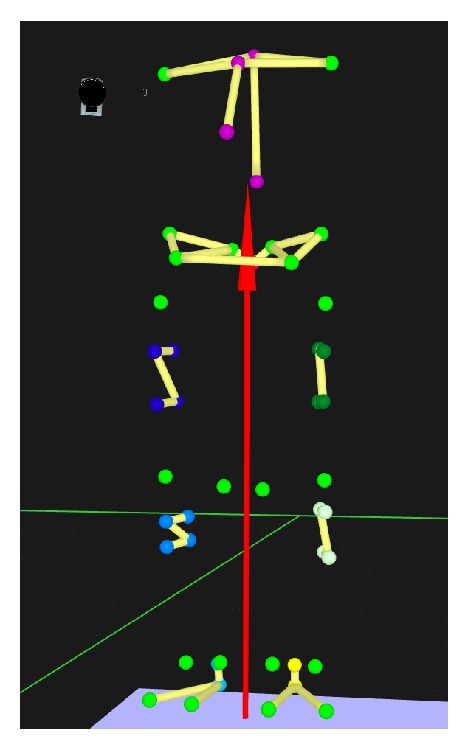
Marker setup used for creating the model. Anatomic markers in bright green. Tracking markers in various colors.

During motion capture, kinematic and kinetic data were collected at 100 Hz as participants walked across the lab floor at a brisk self-selected pace wearing their own comfortable low top walking shoes. Data were collected until 5 successful foot strikes per foot on force plate were obtained. A total of 47 retroreflective markers were used during three-dimensional gait analysis using 8 Oqus 300 infrared cameras (Qualisys AB, Gothenburg, Sweden) synchronized with two AMTI force plates (American Management Technology, Inc., Watertown, USA), embedded into the lab floor. Markers were placed over specific anatomic landmarks of participants in order to define proximal and distal trunk, pelvis, thighs, shanks, and feet as rigid segments, while clusters of 4-5 markers per segment were used for tracking purposes during walking trials (Figure 2). A static measurement was used to define segments and joint centers based on anatomical markers, as well as the relative position of tracking markers and each participant's body mass. Markers were autotracked and labeled using Qualisys Track Manager software while commercial software (Visual3D™, C-Motion, Germantown, USA) was used for further data processing.

Marker and force data were low-pass-filtered using a Butterworth filter with a cut-off frequency at 6 and 20 Hz, respectively. A model template was applied to define body segments and their local reference systems, as well as joint centers. Rigid-body analysis and inverse dynamics postprocessing were conducted to obtain kinematic variables between segments of the lower limbs based on an *x*-*y*-*z* Cardan ordered sequence. Frontal plane trunk motion was calculated with respect to the lab's coordinate system, in order to derive trunk position without the influence of pelvic tilt. Joint moments for the lower limbs were derived by inverse dynamics, resolving the joint moment into the proximal segment, using the software default settings for normalization to body mass (Nm/kg). Specific gait events (IC and TO) were defined by utilizing a force plate data threshold of 15 N, and those events then used for analysis related to the gait cycle, examining trunk lean during stance and specifically at the end of WA. Rate of loading (slope of the moment curve) was calculated as the change in frontal plane external knee joint moment from IC to the first peak KAM over time (seconds) [16]. Data were exported into Microsoft Excel and SPSS statistical software for data compilations and further analysis.

Of OA participants, the knee that was affected was on the left in 8 and on the right in 9 participants, and while 10 participants had unilateral involvement, 7 had some degree of radiographic OA (KL 1–3) on the medial or lateral compartment of the uninvolved side. These baseline data were collected prior to initiation of brace use and are the basis of this investigation. Half of the CTRL group was randomly allocated with a left “involved” and half with a right “involved” knee.

Independent *t*-tests were used to identify group differences in age, BMI, knee ROM, and KOOS. Multivariate analysis was used to identify differences between limbs and groups for strength and temporal-spatial values, trunk lean during WA, and knee joint loading (first peak KAM and rate of loading). A mixed stepwise regression analysis was applied for each group, with loading rate of the KAM during WA being the dependent variable. An initial multiple regression was run to assess the influence of contralateral hip abductor strength values and ipsilateral trunk lean on loading rate at the index knee, also including height and body mass based on biomechanical rationale [17, 26, 27]. Significant predictors of the loading rate were then entered into a sequential multiple linear regression model to determine individual contributions to the explained variance of the dependent variable. Level of significance was set at *p* < 0.05.

## 3. Results

No differences were found for mean age, height, mass, BMI, or strength between groups (Table 1). A significant deficit of knee ROM was evident on the involved side of OA participants as reflected by a group by limb interaction (*F* = 11.824, *p* = 0.002). Moreover, the CTRL group scored significantly higher on all KOOS subscales than the OA group, reflecting expected differences in self-reported knee related symptoms, functional abilities, and quality of life (*p* < 0.001).

No differences were found for mean (SD) walking speed (*p* = 0.316), between OA (1.58 (0.22) m/s) and CTRL (1.65 (0.16) m/s) groups. Duration of stance and swing for each limb's gait cycle (GC) is presented in Table 2 as duration (time (sec)) and relative to the GC (% GC). No differences were found between groups for GC duration and both groups demonstrated interlimb symmetry. However, the OA group demonstrated prolonged stance and a shortened swing phase compared to controls (interaction; *F* = 20.283, *p* < 0.001) and the difference in stance was mostly due to a significantly longer double-limb support phase during WA (*F* = 15.323, *p* < 0.001).

**Figure 3:**
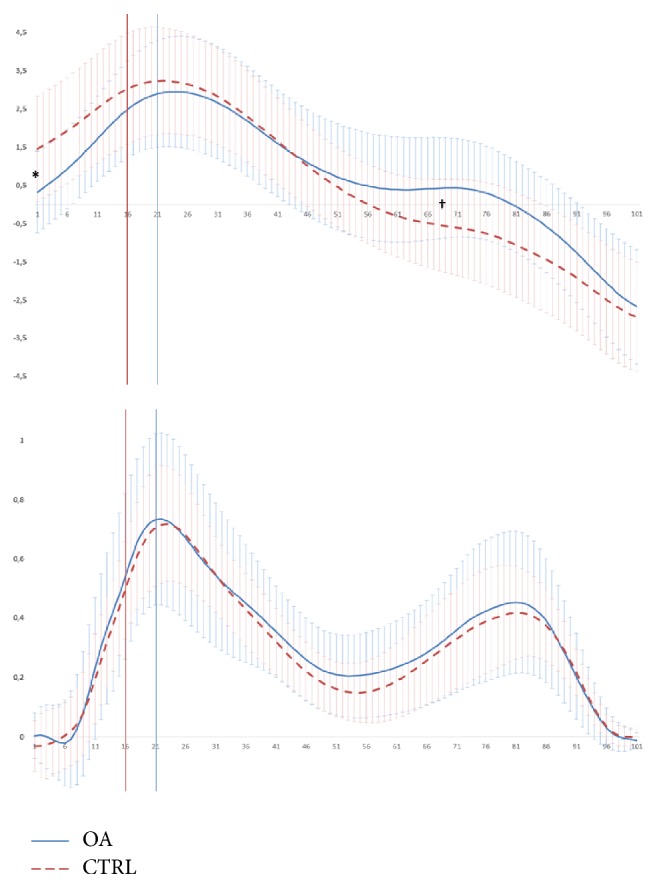
Mean frontal plane trunk angle (degrees), above, and frontal plane knee moment (Nm/kg), below, during the stance phase of the gait cycle (normalized to 101 data points). Trunk angle values are positive for leaning towards the stance side and negative for leaning towards the swing side. Vertical lines indicate end of each group's WA. Significant group difference of trunk angle at IC (^*∗*^*p* < 0.001) and timing of trunk lean transition (^†^*p* < 0.01).

No main effects of group or limb and no interaction with respect to the magnitude of the first peak KAM were found, but for rate of loading a significant main effect of group was found due to differences (*F* = 6.205; *p* = 0.019; Figure 3 and Table 3).

A significant interaction was seen due to group differences in the magnitude of trunk lean at the beginning but not at the end of WA (*F* = 7.136; *p* = 0.012, Figure 3). Post hoc tests revealed that at IC the CTRL group had already initiated trunk lean towards the stance limb (mean (SD) 1.4° (1.4°)), whereas the OA group's trunk position was vertical (0.0° (1.0°); *p* < 0.001). This was further reflected in an earlier transition of CTRL group from a lean towards the stance limb, moving towards the contralateral limb during midstance, which happened at a mean (SD) of 59 (17)% of the stance phase compared to a significantly delayed transition at 71 (21)% of stance for OA participants (*F* = 10.186; *p* = 0.004, Figure 3). The overall group difference in mean peak trunk lean values was only 0.15° (n.s.).

Multiple regression analysis demonstrated that a model that included ipsilateral frontal plane trunk angle at the end of WA, contralateral hip abductor strength, and BMI as independent variables best explained the rate of loading at the index knee. The resulting model demonstrated significance (*F*(3,13) = 15.486, *p* < 0.001, adjusted *R*^2^ = .731) and indicated that each factor added significantly to the model towards lowering the rate of loading (trunk angle (Beta = .635; *p* = 0.001), contralateral hip strength (Beta = .722; *p* < 0.001), and BMI (Beta = .590; *p* = 0.001)). The same model was not successful in predicting ipsilateral PKAM (*p* = 0.067) or in predicting loading rate (*p* = 0.455) or PKAM (*p* = 0.333) on the contralateral side of OA participants or at either knee of controls (*p* > 0.5 for both).

## 4. Discussion

The main results of the study demonstrated that persons with moderate medial knee OA exhibit different movement patterns for frontal plane trunk lean compared to healthy controls. This strategy may assist in maintaining the KAM for this group to levels similar to that of controls during walking. Moreover, the rate of frontal plane knee joint loading during WA may be mediated, in part, by the contralateral limb's abductor strength.

Self-reported measures reflected distinct group differences in symptoms, function, and quality of life as was expected between the knee OA group and healthy controls, whereas other parameters including body size and hip abductor strength were not different. The OA group had a predominantly unilateral involvement with significant ROM deficits of the involved knee. However, although the group demonstrated clear differences in gait patterns when compared with CTRL group, interlimb differences were not evident for temporal-spatial parameters, trunk lean, or knee joint loading values within the OA group.

When temporal-spatial values were observed, the prolonged stance of OA participants was mostly due to a longer WA period, during which a transition is being made from double- to single-limb support. This is in agreement with previous reports [28] and could reflect a pain avoidance strategy by trying to shorten the time in single stance, as this would be the period where the GRF vector is directed more medially and involves greater loading onto the medial compartment than during double stance. The OA group maintained double stance until the first peak KAM while controls were at that point in single stance. Notably, peak KAM values did not capture kinetic differences between the two groups, whereas the rate of loading did. The rate of loading has been implicated in a more rapid rate of degeneration [16]. In addition to a slightly (n.s.) slower gait speed, a significantly prolonged WA for the OA group was coupled with delayed transition of trunk motion, whereby trunk lean towards the stance limb generally started after the initiation of the double stance period. This may represent a more cautious transition of weight and explain the slower rate of loading at the knee seen in the OA group. This, in turn, may contribute to maintaining the first peak KAM to lower values than what might otherwise have been the case for the OA group and thereby positively influence pain levels.

The magnitude of trunk lean has typically been reported as the peak value of the frontal plane motion, with the peak typically being less than 1° greater than that found at the first peak KAM of the knee and occurring somewhat later [13]. Peak values of trunk lean were similar to those previously shown for OA cohorts [6, 13, 29]. Group differences found at IC represent the altered timing of frontal plane trunk motion and were not found at the end of WA or for peak values (Figure 3). This is in accordance with the findings of Hunt et al., where differences in peak frontal plane trunk lean were only found between the group with severe OA and others (with mild or moderate OA and controls) [6]. A position of greater trunk lean at the end of WA was associated with a slower rate of frontal plane knee joint loading at the involved knee in our OA group. As noted previously, this likely reflects the effects of prolonged period of double stance during WA.

Intuitively one might expect that hip abductor strength deficiencies, which are recognized in the OA population [19], would result in less eccentric control, a more rapid contralateral pelvic drop with a resulting greater rate of loading onto the contralateral limb during WA. The results of the present study support this, as greater hip abductor strength on the uninvolved side was an independent predictor of lower rates of frontal plane knee joint loading of the affected knee in the OA group. A cross-sectional study of an OA cohort [30] showed a strong positive association between higher hip abductor strength and better functional performance. A recent systematic review [31] found that strengthening programs for hip abductor muscles improved strength and self-reported scores whereas no change in peak KAM values occurred. Investigating possible effects of hip strengthening exercise on frontal plane knee joint loading rate may cast a light on this relationship.

All participants were male with K-L grade 2 and 3, which may limit the external validity of the study, as sex dependent biomechanical differences are recognized during gait [32] and findings might differ for those with more severe OA (K-L grade 4) [6]. An “a priori” power analysis was not performed, but the observed power for the interactions found was 73% for trunk angle and greater than 90% for temporal-spatial parameters. The secondary regression analysis may suffer from the low number of participants, in particular for the control group, but nonetheless statistical significance was achieved for the affected side of the OA group.

## 5. Conclusion

In order to maintain KAM levels near normal levels, persons with moderate knee OA may benefit from strategies that lower the rate of knee joint loading. Part of this strategy may be achieved by prolonged double stance during WA and altered timing, not degree, of frontal plane trunk motion to affect the magnitude of knee joint loading. Strength of hip abductors may influence their ability to maintain a prolonged trunk position over the stance limb and provide eccentric control while lowering the pelvis prior to contralateral WA, which may also assist in slowing down the rate of loading and controlling peak KAM values.

## Figures and Tables

**Table 1:** Group mean (SD) and range of values for age, BMI, passive range of knee joint motion (ROM), abductor muscle strength, and scores for each subscale of KOOS.

	CTRL	OA
*Age* (years)	49.7 (7.4); 40–65	50.4 (6.2); 40–59
*BMI* (kg/m^2^)	27.1 (7.4); 22.7–32.8	28.3 (3.1); 23.0–34.6
*INV strength* (kg/BMI)	1.05 (0.29); 0.42–1.64	1.04 (0.28); 0.72–1.69
*UN strength* (kg/BMI)	1.07 (0.27); 0.63–1.60	1.05 (0.25); 0.77–1.81
*INV ROM (degrees)*	140.2 (5.3); 133–150	127.3 (10.2); 107–139^*∗*†^
*UN ROM (degrees)*	139.5 (4.1); 134–147	133.8 (9.6); 106–148
*KOOS-pain*	98.9 (2.2); 94–100	64.1 (16.6); 33–94^*∗*^
*KOOS-symptoms*	95.3 (6.2); 82–100	68.3 (15.7); 46–96^*∗*^
*KOOS-ADL*	99.4 (1.1); 97–100	71.8 (17.4); 43–97^*∗*^
*KOOS-sports/rec*	98.9 (2.1); 95–100	31.5 (22.1); 0–80^*∗*^
*KOOS-QOL*	96.9 (6.3); 81–100	38.4 (16.3); 6–63^*∗*^

BMI: body mass index; INV: involved side; UN: uninvolved side; ROM: passive range of motion; ADL: activities of daily living. ^*∗*^Significant difference between groups (*p* < 0.001). ^†^Significant difference between knees (*p* < 0.01).

**Table 2:** Mean (SD) duration (s) and proportion (%) that participants of each group spent in stance versus swing and specifically during weight acceptance (WA) during each limb's gait cycle (GC).

	CTRL	% GC	OA	% GC
Inv stance	0.62 (0.04)	59.4 (1.7)^†^	0.66 (0.06)	62.9 (2.3)^†^
Uninv stance	0.62 (0.04)	59.9 (1.5)^†^	0.66 (0.06)	63.1 (2.7)^†^
Inv swing	0.42 (0.03)	40.6 (1.7)^†^	0.39 (0.04)	37.1 (2.3)^†^
Uninv swing	0.41 (0.03)	40.1 (1.5)^†^	0.38 (0.04)	36.9 (2.7)^†^
Inv WA	0.10 (0.02)	9.3 (1.5)^*∗*^	0.14 (0.03)	13.0 (2.7)^*∗*^
Uninv WA	0.10 (0.02)	9.9 (1.4)^*∗*^	0.13 (0.03)	12.9 (2.6)^*∗*^

Inv: involved limb; Uninv: uninvolved limb. Significant differences between groups for relative proportion of gait cycle (% GC) spent in stance versus swing (^†^interaction of group by phase; *p* < 0.001). Significant group difference for WA as % GC (^*∗*^main effect; *p* < 0.001).

**Table 3:** Mean (SD) values of the first external peak knee adduction moment (PKAM) and rate of loading during loading response at each knee.

	CTRL	OA
Inv PKAM (Nm*∗*kg^−1^)	0.72 (0.19)	0.74 (0.34)
Uninv PKAM (Nm*∗*kg^−1^)	0.74 (0.19)	0.75 (0.26)
Inv loading rate ((Nm*∗*kg^−1^)*∗*s^−1^)	7.68 (2.78)^*∗*^	5.92 (2.99)^*∗*^
Uninv loading rate ((Nm*∗*kg^−1^)*∗*s^−1^)	7.89 (2.79)^*∗*^	5.65 (2.07)^*∗*^

Inv: involved limb; Uninv: uninvolved limb. Significant group difference for rate of loading (^*∗*^main effect; *p* = 0.019).
